# Liquid biopsy in genitourinary cancers: circulating tumor DNA as a predictive biomarker for treatment selection and resistance monitoring

**DOI:** 10.37349/etat.2026.1002407

**Published:** 2026-10-19

**Authors:** Alcides Chaux, Andrea Paola Britos Gómez

**Affiliations:** University of Campania “Luigi Vanvitelli”, Italy; ^1^Facultad de Ciencias de la Salud, Universidad Central del Paraguay, Ciudad del Este 7000, Paraguay; ^2^ChauxLab Institute, Asunción 1230, Paraguay

**Keywords:** circulating tumor DNA, liquid biopsy, genitourinary cancers, predictive biomarker, castration-resistant prostate cancer, urothelial carcinoma, renal cell carcinoma, minimal residual disease

## Abstract

Background: Genitourinary (GU) cancers—prostate, urothelial, and renal cell carcinomas (RCCs)—are a leading source of oncologic morbidity and mortality. Tumor heterogeneity and evolving resistance limit static tissue biopsies, requiring real-time, non-invasive biomarkers. Circulating tumor DNA (ctDNA) captures the tumor’s mutational landscape in real time and has emerged as a candidate biomarker for treatment selection and resistance monitoring in GU malignancies. Methods: An integrative review was conducted by two independent reviewers following the Whittemore and Knafl integrative review framework, informed by PRISMA 2020 conventions. Searches covered PubMed/MEDLINE, Cochrane Central Register of Controlled Trials (CENTRAL), Embase, and Web of Science (January 2020–April 2026). Screening and extraction were performed independently by both reviewers, with disagreements resolved by consensus. After deduplication, 118 records were screened, 39 assessed in full text, and 15 studies met inclusion criteria, stratified by cancer site. Results: ctDNA functions primarily as a prognostic and treatment-monitoring biomarker across GU malignancies, with predictive utility for treatment selection well established in muscle-invasive bladder cancer (MIBC) and still maturing elsewhere. In castration-resistant prostate cancer (CRPC), ctDNA tumor fraction (TF) at enzalutamide cycle 3 independently predicts progression-free and overall survival (OS; median OS 16.0 vs. 22.1 months, ctDNA-detected vs. -undetected with discordant prostate-specific antigen). In MIBC, post-cystectomy ctDNA identifies relapse with 94% sensitivity and 98% specificity, and a ctDNA-stratified IMvigor010 analysis reveals an OS benefit from adjuvant atezolizumab [hazard ratio (HR) 0.59] in ctDNA-positive patients only. In RCC, the least mature stratum, cfDNA quantity, *TP53* mutation, TF, and *BAP1* status are associated with survival on systemic therapy. In upper tract urothelial carcinoma (UTUC), perioperative ctDNA fraction predicts recurrence-free survival, with clonal haematopoiesis accounting for a notable share of false positives. Conclusion: ctDNA is a molecularly grounded biomarker with the strongest evidence in metastatic CRPC (mCRPC) and MIBC, an expanding but preliminary base in RCC, and limited capacity, outside MIBC, to guide treatment selection.

## Introduction

Genitourinary (GU) cancers collectively account for a disproportionate share of the global cancer burden. Prostate cancer (PC) remains the most commonly diagnosed non-cutaneous malignancy among men, with an estimated 1.4 million incident cases annually worldwide. Bladder and urothelial carcinomas (UCs) are responsible for approximately 573,000 new diagnoses per year, and renal cell carcinoma (RCC) contributes an additional 431,000 cases. Together, these three entities generate a substantial and growing demand for precision diagnostic and therapeutic tools [1].

A fundamental limitation in the clinical management of GU cancers is the inherent inadequacy of static tissue biopsy. A single biopsy captures a spatially and temporally restricted genomic snapshot, failing to represent the clonal architecture of disseminated or metastatic disease. In PC, where 90% of metastatic spread localizes to bone, obtaining tissue is both technically challenging and logistically prohibitive. In UC, significant discordance between primary tumors and metastatic sites has been documented, undermining precision medicine strategies anchored exclusively to primary tumor profiling [2, 3]. In RCC, the high degree of intratumoral heterogeneity further confounds tissue-based biomarker interpretation.

Liquid biopsy has emerged as a transformative paradigm in oncology, offering non-invasive, repeatable access to tumor-derived analytes circulating in body fluids. Among these analytes, circulating tumor DNA (ctDNA)—short, apoptosis- and necrosis-derived fragments of tumor genomic DNA released into the peripheral circulation—has attracted the greatest translational momentum. The short plasma half-life of ctDNA, estimated between 15 minutes and 2 hours, enables near-real-time reflection of tumor dynamics under therapeutic pressure. Moreover, ctDNA captures information from multiple tumor sites simultaneously, affording a system-level view of clonal heterogeneity that no single biopsy can provide [2].

The clinical utility of ctDNA spans multiple domains: early detection, prognosis, minimal residual disease (MRD) assessment, treatment response monitoring, and the identification of acquired resistance mutations. In GU oncology, ctDNA has progressed from exploratory biomarker studies toward biomarker-guided clinical trial integration. The FDA approval of the FoundationOne Liquid CDx assay for the identification of *BRCA1*/*BRCA2* mutations in PC patients eligible for rucaparib represents a landmark regulatory milestone, establishing liquid biopsy as a clinically actionable companion diagnostic [4].

Despite this progress, critical gaps remain. The predictive utility of ctDNA—that is, its capacity to inform prospective treatment selection and not merely retrospective prognosis—requires systematic evaluation across the GU cancer spectrum. This integrative review synthesizes current evidence on ctDNA as a predictive biomarker for treatment selection and resistance monitoring in prostate, urothelial, and RCCs, with the objective of consolidating a rigorous evidence base to guide clinical implementation and future trial design.

## Materials and methods

### Study design and methodological framework

This study is an integrative review conducted according to the methodological framework proposed by Whittemore and Knafl [5], informed by PRISMA 2020 reporting conventions [6] adapted to the integrative-review design, and conducted throughout all stages by two reviewers (A.C., A.P.B.G.). The integrative review design was selected for its capacity to synthesize diverse types of evidence—including experimental, observational, and theoretical studies—thereby enabling a comprehensive appraisal of the multi-paradigmatic literature on ctDNA in GU oncology. This design permits inclusion of narrative and integrative reviews alongside primary studies, a scope explicitly excluded from formal systematic reviews, and is reported as such throughout this manuscript; no PROSPERO registration was sought, consistent with standard practice for integrative reviews, which by definition lack a single a priori quantitative protocol amenable to prospective registration.

### Search strategy and information sources

A systematic search was performed across four primary bibliographic databases: PubMed/MEDLINE, Cochrane Central Register of Controlled Trials (CENTRAL), Embase, and Web of Science. The search period spanned January 2020 to April 2026, a time frame selected to capture the contemporary era of ctDNA clinical translation while excluding a preparatory literature base. Search strategies were constructed using combinations of Medical Subject Headings (MeSH) and free-text terms, organized around three conceptual domains: 1) the analyte of interest (“circulating tumor DNA,” “ctDNA,” “liquid biopsy,” “cell-free DNA”); 2) the tumor types of interest (“prostate cancer,” “urothelial carcinoma,” “bladder cancer,” “renal cell carcinoma,” “Genitourinary cancer”); and 3) the clinical outcomes of interest (“treatment selection,” “predictive biomarker,” “resistance monitoring,” “treatment response,” “minimal residual disease”). Boolean operators AND/OR were applied to combine term clusters. The integrity and contextual classification of retrieved references were subsequently verified using SCITE.ai, employed exclusively as a bibliometric validation tool and not as a primary literature source. The four databases searched (PubMed/MEDLINE, CENTRAL, Embase, Web of Science) are indexed, non-predatory sources; no additional exclusion filter for predatory journals was required beyond restricting retrieval to these curated indices. The January 2020 start date was selected to capture the period following the first clinically oriented ctDNA companion-diagnostic validation studies in GU oncology, avoiding an earlier exploratory-technology literature base of limited contemporary clinical relevance; the full per-database search strings are provided in Supplementary material. All classifications and inclusion/exclusion judgments derived from SCITE.ai outputs were reviewed and confirmed manually by both reviewers before incorporation; the tool was used solely as a secondary verification aid and did not replace reviewer judgment at any stage, in accordance with the journal’s policy on artificial intelligence (AI)-assisted tools.

### Eligibility criteria

Studies were eligible for inclusion if they met the following criteria: 1) published in English in peer-reviewed journals or as preprints of sufficient methodological quality between January 2020 and April 2026; 2) reported original or synthesized data on ctDNA as a biomarker in PC, UC (bladder or upper tract), or RCC; 3) addressed at least one of the primary outcomes—treatment selection, treatment response prediction, or resistance monitoring; and 4) provided sufficient methodological or quantitative data to allow extraction of a meaningful synthesis. Studies were excluded if they addressed exclusively non-GU cancer types, focused on circulating tumor cells (CTCs) or other liquid biopsy analytes without ctDNA-specific data, or were conference abstracts without associated full-text publication.

### Study selection and data extraction

The selection process followed a two-stage approach conducted independently and in duplicate by both reviewers (A.C., A.P.B.G.). In the first stage, titles and abstracts of all retrieved records were screened independently by each reviewer against the eligibility criteria; discrepancies were resolved by consensus discussion. In the second stage, full texts of candidate studies were retrieved and independently assessed for final inclusion by both reviewers, with disagreements again resolved by consensus; no third-party arbitration was required. Data extraction was performed independently by both reviewers using a standardized extraction form covering study design, cancer type, patient population characteristics, ctDNA detection methodology, primary and secondary outcomes, and key quantitative findings; extracted data were cross-checked between reviewers, and discrepancies were resolved by re-evaluation against the source publication and the eligibility criteria. Full documentation of the screening and extraction process, including reviewer-level decisions at each stage, is maintained and available upon request.

### Quality assessment

Consistent with integrative review methodology, no single risk-of-bias instrument [e.g., the Cochrane Risk of Bias tool (RoB 2), the Risk Of Bias In Non-randomised Studies of Interventions tool (ROBINS-I), and A MeaSurement Tool to Assess systematic Reviews (AMSTAR-2)] was applied uniformly across the heterogeneous study designs included in this synthesis, as such tools are designed for single study-design strata and are not intended to appraise a mixed corpus of primary studies, case series, and narrative or systematic reviews within a single scale. Instead, certainty of evidence was assessed narratively, by cancer-site stratum and clinical domain (prognosis/MRD, treatment monitoring, and predictive/therapy-selection use), considering study design, sample size, prospective versus retrospective ascertainment, and assay maturity; quantitative claims were anchored preferentially in primary cohort and trial data rather than in review-derived estimates, and heterogeneous assays or cancer sites were not pooled into a single quantitative effect. This narrative certainty appraisal is presented alongside the stratified synthesis in the Results section.

## Results

### Characteristics of the included studies

Database searches across PubMed/MEDLINE, CENTRAL, Embase, and Web of Science for the period January 2020 to April 2026 yielded records that, after deduplication, comprised 118 unique records, screened independently by both reviewers at title and abstract level. Following this stage, 39 records were retrieved and assessed in full text (retrieval complete, 39/39); 25 were excluded, most commonly for wrong population (*n* = 13; not a GU-dedicated synthesis or population) or wrong intervention/analyte (*n* = 12; analyte other than ctDNA, e.g., AR-V7 assessed exclusively via CTCs or tissue). The final synthesis comprised 15 studies: 14 identified through the database search and one additional study identified outside the database search (Figure 1).

**Figure 1:**
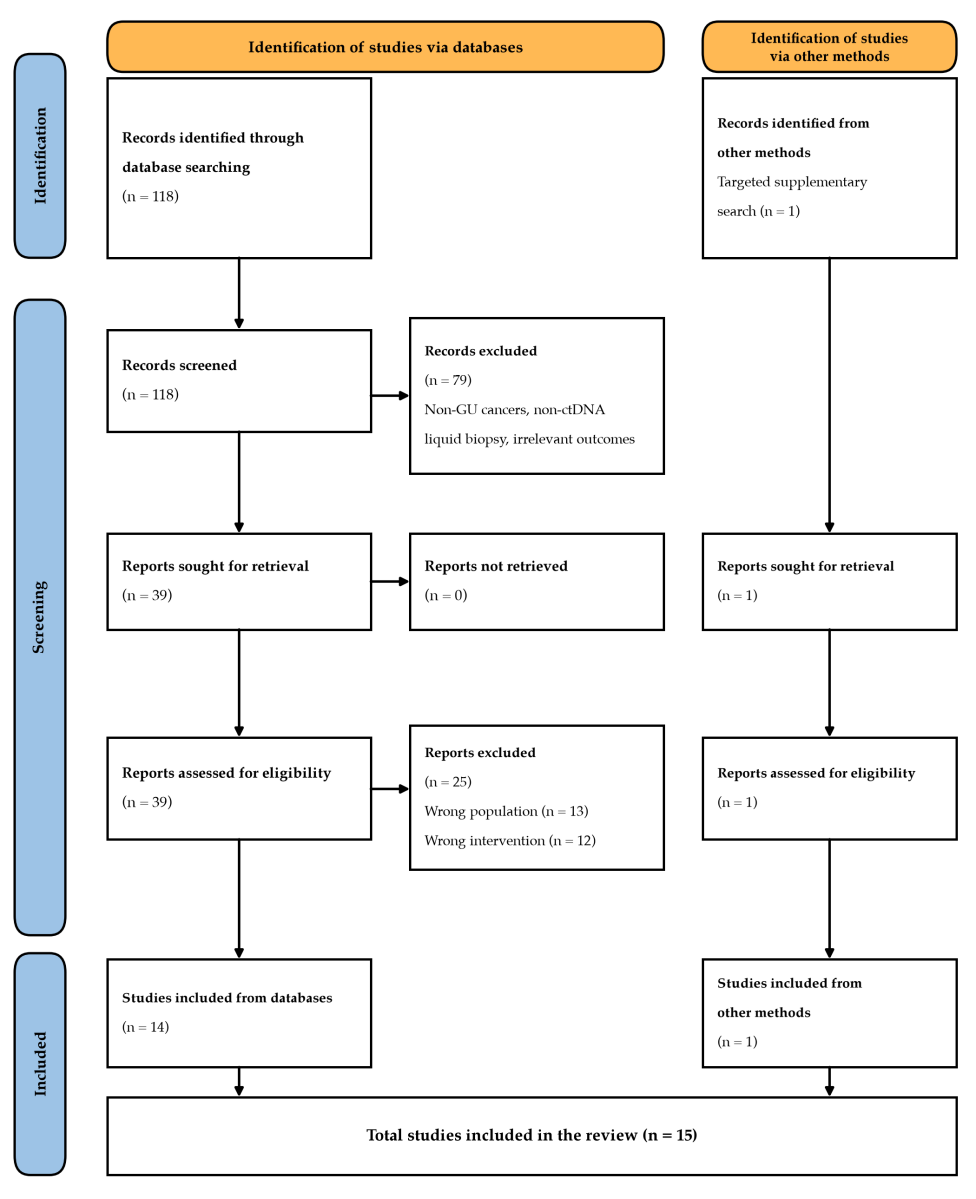
**PRISMA 2020 flow diagram illustrating the study selection process for the integrative review on ctDNA as a predictive biomarker in GU cancers.** Adapted from [6]. CC BY 4.0.

A total of 15 studies met all inclusion criteria and formed the basis of this integrative synthesis. The corpus encompassed original research studies (*n* = 8; 53.3%), narrative reviews (*n* = 5; 33.3%), a systematic review with qualitative synthesis (*n* = 1; 6.7%), and a case series (*n* = 1; 6.7%). Studies were stratified by cancer site: prostate [number of studies per stratum (k) = 4], urothelial/bladder (k = 4), renal (k = 4; see in ctDNA in RCC: emerging evidence and current limitations section), upper tract UC (UTUC; k = 1), and mixed GU cohorts (k = 2). Detailed characteristics of all included studies are presented in Table 1.

**Table 1:** Characteristics of the included studies (*n* = 15).

**Study**	**Design**	**Cancer type**	**Sample/population**	**Key outcome(s)**	**ctDNA method**
Del Re et al., 2021 [7]	Original	Prostate, mCRPC	*n* = 84	AR gain in cfDNA (14.3%) + AR-V7 in exosomes (35.7%) predict primary ARSI resistance; combined-aberration PFS 3.8 vs. 28 months (*P* < 0.0001)	cfDNA + exosomes (ddPCR)
Isebia et al., 2025 [8]	Original, prospective	Prostate, mCRPC	*n* = 196	Baseline GWA-high predicts poor ARSI response (HR 2.63) but not taxanes; early GWA-high predicts both (HR 6.73/2.79)	cfDNA aneuploidy (mFast-SeqS)
Sweeney et al., 2024 [9]	Ph III biomarker (secondary analysis)	Prostate, mCRPC	*n* = 494	ctDNA TF at C3D1 predicts OS; mOS 22.1 vs. 16.0 months (ctDNA-undetected/PSA-not-reduced vs. ctDNA-detected/PSA-reduced; *P* < 0.001)	Tissue-agnostic plasma (FoundationOne Monitor)
Kwan et al., 2022 [3]	Narrative review	Prostate, mCRPC	NR	Implementation pathways: prognosis, monitoring, genotyping; CHIP misattribution risk 15–20%	Multi-platform overview
Lindskrog et al., 2023 [10]	Original, 2-cohort longitudinal	Urothelial, MIBC	*n* = 170 (68 + 102)	Post-RC ctDNA: 94% sensitivity/98% specificity for relapse; NAC-naïve post-RC HR 17.8 (*P* = 0.0002)	Signatera tumor-informed mPCR-NGS
Nawaf et al., 2023 [11]	Narrative review	Urothelial, MIBC	NR	IMvigor010 ctDNA-stratified adjuvant atezolizumab benefit (HR 0.59); TOMBOLA/IMvigor011 ongoing	Signatera + trial landscape
Katsimperis et al., 2025 [12]	Narrative review	Urothelial, MIBC	NR	Tumor-informed vs. agnostic testing; perioperative risk stratification, MRD, bladder-sparing rationale	Multiple
Crocetto et al., 2025 [13]	Systematic review (qualitative)	Urothelial, NMIBC-MIBC	NR	Broader liquid biopsy landscape (urine methylation, CTC, ctDNA); ctDNA one component	Multi-analyte
Del Re et al., 2022 [14]	Original, prospective	Renal, mccRCC	*n* = 48	cfDNA > 0.883 ng/µL + *TP53*mut predict shorter PFS (7.23 vs. NR months; *P* = 0.001) and OS (23.9 vs. NR months; *P* = 0.008)	cfDNA quantity + Oncomine NGS
Kato et al., 2025 [15]	Original, nationwide cohort	Renal, mRCC	*n* = 124	TF < 1.2% → better PFS (HR 0.467); *BAP1* mutation → poor OS (HR 0.487); new mutations at progression in 46.8%	SCRUM-Japan MONSTAR-SCREEN NGS panel
Alam et al., 2025 [16]	Case series	Renal, diverse histologies	*n* = 6	Serial ctDNA guided adjuvant/salvage decisions in all 6 cases; authors note lower sensitivity/NPV in kidney cancers	Signatera
Jikuya et al., 2025 [17]	Narrative review	Renal	NR	Lower ctDNA shedding vs. other tumors; no routine treatment-selection biomarker yet; EVs as complementary analyte	Multi liquid biopsy
Nakano et al., 2022 [18]	Original, prospective	UTUC, perioperative	*n* = 50	Preoperative ctDNA fraction > 2% independently predicts worse RFS; CHIP caused 19% false-positive *TP53* calls	Oncomine Pan-Cancer cfDNA (CHIP-filtered)
Jang et al., 2023 [19]	Original, pilot prospective	Mixed GU (RCC 15, UC 4, PC 1), advanced/ICI	*n* = 20	ctDNA-imaging concordance 83% (15/18); ORR 70%; CNS metastases a discordance blind spot	Signatera tumor-informed
Gerke et al., 2024 [2]	Narrative review	Mixed GU	NR	Cross-site overview of detection/prognosis/monitoring; RCC flagged as highest unmet need	Multiple

This table describes the 15 studies included in the narrative synthesis (see in ctDNA in RCC: emerging evidence and current limitations section). Distribution by design: original research = 8 (53.3%); narrative reviews = 5 (33.3%); systematic review (qualitative) = 1 (6.7%); case series = 1 (6.7%); the IMbassador250 ctDNA biomarker analysis (Sweeney et al. [9]) and the SCRUM-Japan MONSTAR-SCREEN cohort (Kato et al. [15]) are counted as original research. All percentage denominators refer to *n* = 15. mCRPC: metastatic castration-resistant prostate cancer; AR: androgen receptor; AR-V7: AR splice variant 7; ARSI: AR signaling inhibitor; PFS: progression-free survival; ddPCR: digital droplet polymerase chain reaction; GWA: genome-wide aneuploidy; HR: hazard ratio; TF: tumor fraction; C3D1: cycle 3, day 1; mOS: median overall survival; PSA: prostate-specific antigen; NR: not reported; not reached (survival estimates); CHIP: clonal hematopoiesis of indeterminate potential; MIBC: muscle-invasive bladder cancer; Post-RC: post-radical cystectomy; NAC: neoadjuvant chemotherapy; mPCR: multiplex polymerase chain reaction; NGS: next-generation sequencing; MRD: minimal residual disease; NMIBC: non- MIBC; CTC: circulating tumor cell; mccRCC: metastatic clear cell renal cell carcinoma; *TP53*: tumor protein p53; OS: overall survival; mRCC: metastatic renal cell carcinoma; NPV: negative predictive value; EVs: extracellular vesicles; UTUC: upper tract urothelial carcinoma; RFS: recurrence-free survival; GU: genitourinary; RCC: renal cell carcinoma; UC: urothelial carcinoma; PC: prostate cancer; ICI: immune checkpoint inhibitor; ORR: objective response rate; CNS: central nervous system.

### ctDNA in castration-resistant PC (CRPC): tumor fraction (TF), AR-pathway alterations, and treatment monitoring

The most clinically advanced application of ctDNA in GU oncology involves its use as a predictive and monitoring biomarker in metastatic CRPC (mCRPC). PC presents unique challenges for tissue-based genomic profiling: the predominance of bone metastases renders repeat biopsy both technically difficult and clinically hazardous, while tumor heterogeneity between metastatic deposits complicates the representativeness of any single specimen. ctDNA addresses these limitations by providing a composite, systemic view of the evolving genomic landscape [3].

A pivotal contribution to the field was provided by Sweeney et al. [9], who analyzed ctDNA TF data from IMbassador250, a phase III trial evaluating enzalutamide with or without atezolizumab in post-abiraterone mCRPC patients. In 494 evaluable participants, baseline ctDNA TF detection and its dynamics through cycle 3, day 1 (C3D1) were evaluated as biomarkers of clinical outcome. Patients with detectable ctDNA TF at C3D1, irrespective of baseline status, demonstrated significantly shorter radiographic progression-free survival (rPFS) and overall survival (OS) than those without ctDNA detection. Critically, when ctDNA TF and prostate-specific antigen (PSA) response were discordant at C3D1, patients who were ctDNA-undetected but PSA-non-reduced achieved superior OS [median OS (mOS) 22.1 months] relative to those who were ctDNA-detected despite PSA reduction (mOS 16.0 months; *P* < 0.001). This finding established ctDNA TF as an independent and complementary biomarker to PSA in assessing treatment response, with the capacity to identify a clinically distinct patient subgroup inadequately characterized by conventional PSA kinetics alone.

Circulating markers of the androgen receptor (AR) pathway offer a mechanistically grounded, ctDNA/cfDNA-based approach to treatment selection in CRPC. AR-V7, a constitutively active splice variant lacking the ligand-binding domain of full-length AR, confers primary resistance to the AR signaling inhibitors (ARSIs) enzalutamide and abiraterone; detection of this pathway via cell-free DNA and exosomal RNA—rather than via CTCs or tissue, the more classical detection routes—is the focus of this ctDNA-specific synthesis. Del Re et al. [7] demonstrated in a retrospective cohort of 84 chemotherapy-naive mCRPC patients starting first-line abiraterone or enzalutamide that AR copy-number gain in cfDNA (detected in 14.3% of patients) and AR-V7 detection in circulating exosomal RNA (35.7%) each independently predicted primary ARSI resistance; patients with both aberrations had markedly shorter progression-free survival (PFS; 3.8 vs. 28.0 months) and OS (6.1 months vs. not reached; *P* < 0.0001 for both) than patients with neither. Complementary cfDNA-based approaches to treatment selection in mCRPC include genome-wide aneuploidy (GWA) scoring: in a prospective cohort, a high cell-free DNA aneuploidy score at baseline and at an early on-treatment timepoint was associated with poorer response to ARSIs, and the early-timepoint score also predicted poorer response to taxanes [8].

AR-V7 status can also be assessed via CTC-based platforms, most notably the Epic Sciences enumeration assay, independently validated as a prognostic biomarker of OS in men with progressing mCRPC starting an ARSI, across a discovery cohort (Memorial Sloan Kettering, *n* = 171) and the multicenter PROPHECY validation trial (*n* = 107) [20]. CTC- and cfDNA/exosome-based approaches to AR-pathway assessment are complementary rather than competing technologies: CTC platforms offer single-cell resolution and established clinical validation in this specific application, whereas cfDNA/exosome-based assays offer higher throughput, do not depend on CTC capture efficiency, and can be co-analyzed alongside other ctDNA-based biomarkers (TF, DNA damage repair alterations) from a single blood draw. This CTC-based literature is not itself synthesized in this review, whose scope is specifically ctDNA/cfDNA, but is noted here as the relevant comparator technology.

### ctDNA in muscle-invasive bladder cancer (MIBC): MRD, adjuvant therapy guidance, and immunotherapy response

In MIBC, ctDNA has been validated across the perioperative and metastatic continuum as a biomarker of MRD, treatment response, and relapse prediction. The biological plausibility is well-established: ctDNA concentrations correlate with tumor burden, and the short plasma half-life of these fragments allows rapid reflection of changes induced by surgical or systemic interventions [2, 12].

Lindskrog et al. [10] reported landmark longitudinal ctDNA data from two independent MIBC cohorts with median follow-up exceeding five years. In 68 neoadjuvant chemotherapy (NAC)-treated patients, post-cystectomy ctDNA assessment identified metastatic relapse with 94% sensitivity and 98% specificity. ctDNA dynamics during NAC were independently associated with patient outcomes when adjusted for pathological downstaging [hazard ratio (HR) 4.7; *P* = 0.029]. In a separate NAC-naïve cohort of 102 patients, preoperative ctDNA positivity predicted significantly inferior recurrence-free survival (RFS; HR 3.4; *P* = 0.0005) and was an even stronger post-cystectomy prognostic factor (HR 17.8; *P* = 0.0002). Biologically, baseline ctDNA positivity was associated with the basal/squamous molecular subtype and enrichment of epithelial-to-mesenchymal transition gene sets. These findings collectively establish ctDNA as a superior prognostic tool to conventional pathological staging in MIBC.

The predictive utility of ctDNA in guiding adjuvant immunotherapy decisions in MIBC was substantiated by data from the IMvigor010 trial [21]. A post-hoc ctDNA analysis demonstrated that patients with detectable ctDNA after radical cystectomy derived meaningful disease-free survival benefit from adjuvant atezolizumab, whereas ctDNA-negative patients did not show equivalent advantage [22]. Moreover, ctDNA clearance during treatment occurred more frequently with atezolizumab than with observation (18% vs. 4%), consistent with an on-treatment effect on molecular residual disease [22]. These data position ctDNA as a predictive, not merely prognostic, instrument for adjuvant therapy selection in MIBC. This effect was formally confirmed in the trial’s ctDNA-stratified OS analysis [23], which reported an OS HR of 0.59 (95% CI 0.42–0.83) favoring atezolizumab specifically among ctDNA-positive patients, establishing IMvigor010 ctDNA status as a predictive, not merely prognostic, biomarker for adjuvant immunotherapy benefit in this population.

Quantitative claims regarding the ABACUS and NABUCCO trials are not included in this synthesis because they could not be traced to a verifiable primary source (see Discussion, limitations).

Nawaf et al. [11] reviewed ctDNA-based MRD detection in the context of adjuvant therapy decision-making for MIBC. TOMBOLA (NCT04138628) and IMvigor011 are ongoing prospective trials designed to test whether withholding or administering adjuvant atezolizumab according to postoperative ctDNA status improves outcomes relative to standard management; mature efficacy results were not yet available in the sources captured by this review’s search (through April 2026) and are therefore not quoted here pending peer-reviewed publication. Crocetto et al. [13] provided a complementary systematic synthesis of liquid biopsy applications in bladder cancer, contextualizing ctDNA within a broader multi-analyte framework that includes CTCs and urinary exosomes; because this synthesis spans multiple analytes beyond ctDNA, its contribution to the present review is limited to the ctDNA-specific findings it reports.

The pilot study by Jang et al. [19] extended these observations to the metastatic GU setting, prospectively enrolling 20 patients with advanced GU malignancies—15 with RCC, 4 urothelial, and 1 prostate—receiving immune checkpoint inhibitors (ICIs). Using the tumor-informed Signatera multiplex polymerase chain reaction (mPCR)-based next-generation sequencing (NGS) assay, longitudinal ctDNA analysis demonstrated an overall concordance of 83% (15 of 18 evaluable patients) between ctDNA dynamics and radiographic response. The overall objective response rate was 70%. Among three discordant cases, two involved central nervous system metastases, a known blind spot for systemic ctDNA assays, underscoring the importance of integrating ctDNA with anatomic imaging. This study provides proof-of-concept for ctDNA-guided ICI monitoring across GU cancer types.

Beyond MRD and prognosis, ctDNA-based molecular subtyping—including detection of *FGFR3* alterations—has been proposed as a route to non-invasive treatment stratification in UC, linking luminal-papillary tumors to FGFR-directed therapy and basal/squamous tumors to immunotherapy or chemotherapy [13]. However, no study meeting this review’s ctDNA-specific eligibility criteria reported a quantitatively verifiable analysis of ctDNA-based *FGFR3* detection performance or of erdafitinib response stratified by ctDNA *FGFR3* status; this application is therefore noted as an emerging direction rather than as established evidence, and specific response-rate figures are not cited here, pending identification of a verifiable primary source.

### ctDNA in RCC: emerging evidence and current limitations

Among the three principal GU cancer types, RCC represents the domain where ctDNA evidence is least mature but where clinical need is most acute. Liquid biopsy offers a particularly compelling solution in RCC given the challenges of percutaneous renal mass biopsy in certain anatomical locations. The high prevalence of morphologically heterogeneous tumors and the frequent necessity of distinguishing clear cell from non-clear cell RCC subtypes for systemic therapy selection also contribute to the utility of liquid biopsy [17].

Clear cell RCC is characterized by *VHL* gene inactivation in up to 90% of cases, often accompanied by concurrent alterations in *PBRM1*, *SETD2*, *BAP1*, and *KDM5C*. These mutations generate tumor-specific ctDNA fragments detectable in plasma, albeit at levels typically lower than those observed in prostate or UCs, partly because RCC frequently lacks the high tumor mutational burden seen in urothelial malignancies [15, 17]. *PBRM1* mutations have been proposed as predictive biomarkers of benefit from ICI therapy in metastatic RCC (mRCC), based on retrospective analyses of tumor tissue; whether ctDNA-based *PBRM1* detection achieves equivalent predictive accuracy awaits prospective validation.

Del Re et al. [14] quantified plasma cfDNA amount and tumor protein p53 (*TP53*) mutational status in 48 patients with metastatic clear cell RCC (mccRCC) receiving first-line immunotherapy (ipilimumab-nivolumab) or VEGFR-tyrosine kinase inhibitors: cfDNA concentration above 0.883 ng/µL was associated with shorter PFS (7.23 vs. not-reached months; *P* = 0.001) and OS (23.9 vs. not-reached months; *P* = 0.008), and *TP53*-mutant cfDNA independently predicted shorter PFS (*P* = 0.04), with the combination of high cfDNA and *TP53* mutation identifying the poorest-prognosis subgroup. In a considerably larger, more recent nationwide genomic profiling study, Kato et al. [15] analyzed serial ctDNA in 124 patients with mRCC enrolled in the SCRUM-Japan MONSTAR-SCREEN project: baseline TF below 1.2% was associated with significantly better first-line PFS (HR 0.467, 95% CI 0.229–0.979), *BAP1* mutational status at baseline predicted poorer OS (HR 0.487, 95% CI 0.322–0.736), and 46.8% of patients acquired new ctDNA mutations at disease progression, a finding linked to shorter time to progression. This larger prospective cohort strengthens the primary-study basis for ctDNA prognostic use in RCC, which had previously relied on smaller retrospective cohorts and case series. Alam et al. [16] described a case series of six patients with diverse renal malignancies—including clear cell RCC, papillary RCC, translocation RCC, malignant angiomyolipoma, and squamous cell carcinoma of the renal pelvis—in whom serial ctDNA monitoring informed clinical decisions in adjuvant and metastatic settings in all six cases; the authors explicitly noted lower assay sensitivity and negative predictive value in kidney cancers relative to other tumor types, consistent with the lower tumor mutational burden and lower ctDNA shedding characteristic of RCC noted throughout this synthesis.

Jikuya et al. [17] provided a collaborative narrative synthesis of translational RCC research, situating liquid biopsy—including ctDNA, cell-free DNA quantity, and extracellular vesicles—within the broader landscape of RCC biomarker development, and explicitly noting that ctDNA detection rates in RCC remain lower than in other solid tumors under older digital PCR-based assays, with NGS platforms improving sensitivity for low tumor-fraction detection. Gerke et al. [2] similarly identified RCC as the GU cancer type with the greatest unmet need for ctDNA biomarker validation. Taken together, the RCC evidence base—now including one adequately powered primary cohort (Kato et al. [15], *n* = 124) alongside smaller original studies and case-level data—supports cfDNA quantity, *TP53* mutation, TF, and *BAP1* mutational status as prognostic and monitoring biomarkers, but does not yet support routine ctDNA-guided treatment selection in RCC outside investigational settings; alternative and complementary biomarkers under active investigation, including DNA methylation-based predictors of systemic-therapy sensitivity, are addressed in the Discussion.

### ctDNA in UTUC

UTUC is addressed here as a distinct stratum. Nakano et al. [18] prospectively studied 50 patients with untreated UTUC using perioperative plasma ctDNA (pan-cancer NGS panel) with matched buffy-coat sequencing for clonal hematopoiesis of indeterminate potential (CHIP) exclusion. ctDNA was detected in 23/50 (46%) of the overall cohort and 17/43 (40%) of localized cases; preoperative ctDNA fraction greater than 2% was the only independent preoperative risk factor for worse RFS among the factors assessed, and early postoperative ctDNA positivity was associated with worse RFS, consistent with MRD. Critically, 19% of detected *TP53* mutations were false positives attributable to CHIP, underscoring the magnitude of this analytic confounder in a GU-specific population and reinforcing the buffy-coat-matched sequencing approach as good practice for ctDNA-based UTUC studies. Serial ctDNA also tracked systemic therapy response in metastatic UTUC in this cohort. This single-cohort evidence base is mechanistically informative but requires prospective, multi-institutional validation before ctDNA can inform perioperative treatment decisions in UTUC.

### Detection technologies and clinical implementation considerations

The clinical deployment of ctDNA biomarkers requires robust, standardized, and analytically validated assay platforms. Three principal methodological approaches dominate the contemporary landscape. Digital droplet polymerase chain reaction (ddPCR) offers high sensitivity for the detection of predetermined mutations but is limited in scope to a restricted set of predefined targets. NGS panels, including commercially available platforms such as Guardant360 and FoundationOne Liquid CDx, provide broader genomic coverage and are capable of detecting multiple classes of genomic alterations—point mutations, copy number variations, structural rearrangements, and microsatellite instability—simultaneously. Tumor-informed mPCR-NGS assays, exemplified by Signatera, are designed around patient-specific tumor mutation profiles derived from tissue sequencing and achieve the highest sensitivity for MRD detection in the postoperative setting [10, 19].

A critical technical challenge across all platforms is the confounding effect of CHIP. In this case, age-related somatic mutations in hematopoietic stem cells generate ctDNA-like fragments in plasma that are indistinguishable from tumor-derived signals without matched germline sequencing. Failure to account for CHIP can introduce false-positive findings, particularly in older patients—a demographic that constitutes the majority of GU cancer cases. Pre-analytical standardization, including cell-free DNA collection tube protocols, centrifugation parameters, and storage conditions, also substantially influences ctDNA yield and quality, complicating cross-laboratory comparisons [3]. In UTUC specifically, CHIP accounted for 19% of detected *TP53* mutations misclassified as tumor-derived in a dedicated perioperative cohort, illustrating the magnitude of this analytic risk in a GU-specific population when buffy-coat-matched sequencing is not performed [18].

Urine offers an alternative and complementary liquid biopsy matrix in GU oncology, particularly for bladder and urothelial cancers where direct contact between the tumor and the urinary tract generates high concentrations of tumor-derived DNA. *TERT* promoter mutations, detectable in urine ctDNA with high specificity for bladder cancer, represent a validated molecular marker for surveillance. The complementarity between plasma and urine ctDNA matrices enhances overall biomarker sensitivity, particularly in early-stage and localized disease where systemic ctDNA shedding is low [13].

## Discussion

This integrative review consolidates evidence across 15 studies (see in ctDNA in RCC: emerging evidence and current limitations section) to demonstrate that ctDNA functions as a clinically meaningful prognostic and treatment-monitoring biomarker across four GU cancer strata—prostate, urothelial/bladder, renal, and UTUC—with predictive utility for prospective treatment selection that is well established in MIBC and still maturing elsewhere. The most mature evidence base exists in CRPC and MIBC, where ctDNA has been embedded within phase III trial analyses. The evidence base in RCC remains the least mature of the four strata but is expanding, and UTUC evidence, while limited to a single dedicated cohort, is mechanistically informative regarding CHIP as an analytic confounder.

A terminological distinction is important throughout this discussion. A predictive biomarker indicates whether a specific treatment is more or less likely to benefit a given patient, informing a treatment-selection decision; a prognostic biomarker indicates the expected course of disease regardless of treatment. Most of the evidence synthesized here—ctDNA TF and mutational status as correlates of progression-free and OS—is prognostic and treatment-monitoring evidence rather than predictive evidence in this strict sense. The clearest example of genuinely predictive ctDNA evidence within this synthesis is the IMvigor010 ctDNA-stratified analysis, in which ctDNA-positive status identifies patients who derive a specific OS benefit from adjuvant atezolizumab that ctDNA-negative patients do not [23]—i.e., ctDNA status modifies the expected effect of a specific treatment, the defining feature of a predictive biomarker. Where this synthesis uses "predictive" in the broader, looser sense common in the liquid-biopsy literature, this is noted explicitly to avoid conflating the two constructs; this distinction directly bears on which findings are ready to inform treatment-selection decisions today (chiefly, MIBC adjuvant immunotherapy) versus which remain prognostic or monitoring signals awaiting prospective predictive validation (most of the CRPC and RCC evidence).

The clinical stakes of this distinction are illustrated concretely by AR-pathway assessment in mCRPC. Bone is the dominant site of metastatic spread in PC, and repeat bone biopsy for tissue-based AR-V7 testing is frequently not feasible in routine practice because of technical difficulty, procedural risk, and turnaround time incompatible with urgent treatment decisions; a patient beginning to fail an ARSI cannot always wait for a bone biopsy result before a treatment-sequencing decision (ARSI continuation versus transition to taxane chemotherapy) must be made. ctDNA/cfDNA- and exosome-based AR-pathway assessment removes this specific barrier by requiring only a peripheral blood draw. This clinical accessibility advantage must be weighed, however, against cost and infrastructure barriers on the other side of implementation: commercial ctDNA panels (e.g., FoundationOne Liquid CDx, Signatera, Guardant360) carry substantial per-test costs and require reference-laboratory turnaround, and their accessibility varies markedly across health systems and geographic regions, a limitation particularly relevant to resource-constrained settings where the burden of GU malignancies remains high. Formal cost-effectiveness analyses of ctDNA-guided treatment selection in GU oncology remain scarce in the literature captured by this review, and this gap is identified as a priority area alongside the clinical-effectiveness gaps discussed below.

In RCC specifically, where ctDNA shedding is characteristically low, methylation-based cfDNA biomarkers are being explored as a complementary or alternative approach to mutation-based ctDNA detection: a systematic review of 31 studies found that promoter methylation status is associated with treatment response to systemic agents in RCC, although the authors characterize this evidence as still weak and in need of prospective validation [24]. Methylation-based assays may partially overcome the low tumor-fraction ceiling that limits mutation-based ctDNA detection in RCC, and their integration alongside the mutation-based findings summarized in ctDNA in RCC: emerging evidence and current limitations section represents a concrete direction for future RCC-focused liquid biopsy research. More broadly, priority areas for future investigation identified by this synthesis include: (1) a prospective, randomized trial testing ctDNA TF-guided treatment switching (continue ARSI vs. transition to taxane) at C3D1 in mCRPC, using the discordant ctDNA/PSA subgroup identified by Sweeney et al. [9] as the enrichment population; (2) maturation and reporting of the ongoing TOMBOLA and IMvigor011 trials testing ctDNA-guided adjuvant therapy de-escalation and escalation in MIBC; (3) an adequately powered prospective cohort combining mutation-based and methylation-based cfDNA assays in RCC, stratified by histologic subtype; and (4) formal cost-effectiveness modeling of ctDNA-guided treatment selection in at least one GU cancer site with mature predictive evidence (MIBC).

The finding that ctDNA TF dynamics at C3D1 of enzalutamide therapy provide prognostic information that is discordant with, and in a defined subgroup diverges from, PSA response has clinically relevant implications for treatment monitoring in mCRPC [9], without implying that ctDNA TF should replace PSA as a routine monitoring tool at this stage. PSA, the longstanding cornerstone of PC treatment monitoring, is susceptible to flare phenomena and is uninformative in a subset of patients with neuroendocrine differentiation. ctDNA TF provides an orthogonal, molecularly grounded measurement that captures tumor viability independently of PSA secretory function, potentially enabling earlier identification of patients unlikely to benefit from continued ARSI therapy; prospective validation of ctDNA TF-guided treatment switching, however, remains an important evidence gap before this orthogonal signal can inform routine practice.

The convergence of AR-pathway signals detected via cfDNA (AR copy-number gain) and via exosomal RNA (AR-V7) in the same patient cohort [7] strengthens the mechanistic coherence of AR-pathway liquid biomarkers as treatment-informative signals in CRPC, complementing the independently validated CTC-based Epic Sciences AR-V7/enumeration platform [20] discussed in ctDNA in castration-resistant PC (CRPC): tumor fraction (TF), AR-pathway alterations, and treatment monitoring section. Prospective randomized data demonstrating that AR-pathway-guided treatment allocation (ARSI-to-taxane transition) improves hard outcomes remain an important evidence gap common to all three detection modalities (cfDNA, exosomal RNA, and CTC).

In MIBC, the hypothesis that ctDNA-negative status after radical cystectomy could identify patients at sufficiently low recurrence risk to permit watchful waiting rather than adjuvant immunotherapy is being tested prospectively in TOMBOLA and IMvigor011 [11]; if confirmed, ctDNA-negativity criteria could reduce the number of patients requiring adjuvant atezolizumab, with corresponding reductions in treatment-related toxicity and healthcare costs. At present, however, the strongest predictive evidence for adjuvant therapy selection in MIBC is the ctDNA-stratified OS benefit demonstrated in IMvigor010 (HR 0.59 favoring atezolizumab in ctDNA-positive patients) [23], while the watchful-waiting strategy for ctDNA-negative patients remains investigational pending mature prospective trial results.

The high sensitivity (94%) and specificity (98%) of post-cystectomy ctDNA for metastatic relapse detection reported by Lindskrog et al. [10] are particularly remarkable and merit contextualization. These performance metrics exceed those of conventional imaging modalities for early relapse detection and are achieved with a lead time of several months before radiographic evidence of recurrence emerges. The clinical implication is that ctDNA-positive patients could be entered into salvage clinical trials or receive intensified therapy at a point when disease burden is lowest, and the probability of treatment benefit is highest—a window that conventional imaging surveillance consistently misses.

ctDNA does not operate in isolation within the precision oncology framework. Its clinical impact is maximized when integrated with complementary biomarkers—including tumor mutational burden, microsatellite instability status, PD-L1 expression, and molecular subtype classification—into multiparameter decision models. In UC, for example, the identification of *FGFR3* mutations in ctDNA contextualizes treatment selection within the broader molecular landscape of luminal versus basal tumor subtypes. *FGFR3* alterations have been proposed as immune-cold correlates that may favor FGFR-directed therapy over immune checkpoint inhibition, though this association was not independently verified by a ctDNA-specific primary study within this review’s inclusion criteria and is presented as hypothesis-generating [13]. ctDNA-based *FGFR3* monitoring after erdafitinib initiation can then provide early evidence of secondary resistance, enabling timely therapeutic re-evaluation.

Similarly, in CRPC, the integration of ctDNA TF with AR-pathway status and DNA damage repair gene alterations (*BRCA1*/*BRCA2*, *CDK12*)—detectable on the same NGS-based ctDNA platforms described in Detection technologies and clinical implementation considerations section—offers, in principle, a multi-dimensional resistance profile to guide the rational sequencing of ARSIs, taxanes, PARP inhibitors, and immunotherapy agents; however, no study meeting this review’s eligibility criteria reported prospective clinical outcomes from such a combined ctDNA panel in a GU-specific cohort, and this integration is presented as a conceptual framework for future prospective validation rather than as established evidence.

AI and machine learning (ML) algorithms are increasingly being explored, across oncology broadly, for extracting prognostic and predictive signals from ctDNA data that may exceed the capacity of conventional statistical models, including integration of ctDNA mutational profiles with radiomic, proteomic, and clinical covariates. Within the GU-specific ctDNA literature captured by this review’s eligibility criteria, however, no study reported a validated AI/ML model applied to ctDNA data in prostate, urothelial, or renal cancer; this application is therefore an investigational direction rather than established evidence at present, and should not be interpreted as an imminent clinical tool.

Various limitations inherent to this integrative review and to the underlying evidence base warrant careful consideration. First, the majority of ctDNA studies in GU oncology to date have been performed as retrospective or post-hoc analyses of prospectively collected biospecimens, often with incomplete ctDNA sampling at all intended time points. These designs, while generating hypothesis-rich data, are subject to selection and attrition biases that may inflate biomarker performance estimates. Prospective, pre-specified ctDNA biomarker studies embedded within interventional trials constitute the gold standard for predictive biomarker validation and remain relatively scarce in GU oncology.

Second, the heterogeneity of ctDNA detection platforms across included studies creates a significant impediment to cross-study quantitative synthesis. The absence of harmonized pre-analytical protocols, standardized variant allele frequency thresholds, and shared bioinformatics pipelines means that ctDNA positivity rates and predictive accuracies reported across studies are not directly comparable. This methodological fragmentation is particularly evident in RCC, where the low ctDNA shedding rate demands ultrasensitive detection technologies that are not uniformly available.

Third, this integrative review is subject to limitations intrinsic to the methodology itself. Whittemore and Knafl’s [5] framework enables the inclusion of diverse study designs, which broadens the evidence base but also introduces heterogeneity in methodological quality; for this reason, no single quantitative appraisal instrument was applied uniformly (see Quality assessment section), and quantitative claims were anchored preferentially in primary cohort and trial data. The inability to perform a formal meta-analysis due to the diversity of outcomes, time points, assay platforms, and cancer types limits the precision of the synthesized conclusions, and heterogeneous assays and cancer sites were not pooled into a single quantitative effect at any point in this synthesis. Publication bias may favor studies with positive findings, potentially overrepresenting the clinical utility of ctDNA relative to studies with null or inconclusive results. Quantitative claims in this synthesis were anchored preferentially to primary trial or cohort publications; a small number of claims that could not be traced to a verifiable primary source are not included (see ctDNA in muscle-invasive bladder cancer (MIBC): MRD, adjuvant therapy guidance, and immunotherapy response section). A related, contemporaneous narrative review addressing ctDNA specifically in urothelial cancers [25] was published after this review’s literature search closed and was not itself eligible for inclusion; it is noted here for transparency, and its scope (urothelial cancer only) differs from the cross-site synthesis presented in this manuscript. Finally, this review’s scope is restricted to prostate, urothelial (bladder and upper tract), and RCCs; penile and testicular malignancies, also classified as GU cancers, were outside the eligibility criteria and are not addressed here, and their considerably smaller ctDNA evidence base merits dedicated future synthesis.

In conclusion, this integrative review demonstrates that ctDNA constitutes a scientifically grounded and clinically promising biomarker across the GU cancer spectrum, with the most compelling—and, in MIBC, genuinely predictive—evidence in mCRPC and MIBC. It also highlights an emerging but still preliminary evidence base in RCC, now strengthened by an adequately powered primary cohort, and a mechanistically informative single-cohort evidence base in UTUC. The integration of ctDNA into prospective trial designs, regulatory companion diagnostic frameworks, and clinical decision algorithms represents the critical translational pathway through which liquid biopsy will transition from a research instrument to a standard-of-care tool. Priority areas for future investigation include prospective randomized trials of ctDNA-guided treatment allocation, analytical standardization of assay platforms, formal cost-effectiveness evaluation, and the development of multiparameter biomarker models—potentially combining mutation- and methylation-based cfDNA analysis—that capture the full complexity of therapeutic resistance in GU oncology.

## Abbreviations

AI: artificial intelligence
AR: androgen receptor
ARSI: androgen receptor signaling inhibitor
AR-V7: androgen receptor splice variant 7
*BAP1*: *BRCA1*-associated protein 1
*BRCA1*: *BRCA1* DNA repair associated
C3D1: cycle 3, day 1
CENTRAL: Cochrane Central Register of Controlled Trials
CHIP: clonal hematopoiesis of indeterminate potential
CNS: central nervous system
CRPC: castration-resistant prostate cancer
CTCs: circulating tumor cells
ctDNA: circulating tumor DNA
ddPCR: digital droplet polymerase chain reaction
EVs: extracellular vesicles
*FGFR3*: fibroblast growth factor receptor 3
GU: genitourinary
GWA: genome-wide aneuploidy
HR: hazard ratio
ICIs: immune checkpoint inhibitors
k: number of studies per stratum
mccRCC: metastatic clear cell renal cell carcinoma
mCRPC: metastatic castration-resistant prostate cancer
MIBC: muscle-invasive bladder cancer
mOS: median overall survival
mPCR: multiplex polymerase chain reaction
mRCC: metastatic renal cell carcinoma
MRD: minimal residual disease
NAC: neoadjuvant chemotherapy
NGS: next-generation sequencing
NR: not reported; not reached (survival estimates)
ORR: objective response rate
OS: overall survival
*PBRM1*: polybromo 1
PC: prostate cancer
PFS: progression-free survival
Post-RC: post-radical cystectomy
PSA: prostate-specific antigen
RCC: renal cell carcinoma
RFS: recurrence-free survival
TF: tumor fraction
*TP53*: tumor protein p53
UCs: urothelial carcinomas
UTUC: upper tract urothelial carcinoma

## Supplementary materials

The supplementary material for this article is available at: https://www.explorationpub.com/uploads/Article/file/1002407_sup_1.pdf.

## Declarations

### Availability of data and materials

This integrative review analyzed published literature only; no new primary data were generated. The included studies are listed in Table 1 and the References and are accessible through the databases listed in the Methods (PubMed/MEDLINE, Cochrane Central Register of Controlled Trials (CENTRAL), Embase, and Web of Science). The per-database search strategies are provided in the Supplementary material. Additional supporting data (screening and extraction records) are available from the corresponding author upon request.

### Copyright

© The Author(s) 2026.
