# Supplementary material for: Liquid biopsy in genitourinary cancers: circulating tumor DNA as a predictive biomarker for treatment selection and resistance monitoring
Source: Explor Target Antitumor Ther. 2026 Oct 19;7:1002407. doi: 10.37349/etat.2026.1002407 (PMC13739405; doi:10.37349/etat.2026.1002407)
Supplement: Supplementary file 1 [file 1002407_sup_1.pdf]

Supplementary Material: Per-Database Search Strategies

Manuscript: Liquid Biopsy in Genitourinary Cancers: Circulating Tumor DNA as a Predictive Biomarker for Treatment Selection and Resistance Monitoring **Manuscript ID:** ETAT-2026-00103

Search strategies were built around three conceptual domains, combined with the Boolean operators AND (between domains) and OR (within domains), as described in Section 2.2 of the manuscript: (1) the analyte of interest, (2) the GU tumor types of interest, and (3) the clinical outcomes of interest. All searches were restricted to January 1, 2020 – April 30, 2026, and to English-language records.

1. PubMed/MEDLINE

```
("circulating tumor DNA"[MeSH Terms] OR "circulating tumor DNA"[tiab] OR "ctDNA"[tiab]
  OR "liquid biopsy"[tiab] OR "cell-free DNA"[tiab] OR "cell free DNA"[tiab])
AND
("prostatic neoplasms"[MeSH Terms] OR "prostate cancer"[tiab]
  OR "urinary bladder neoplasms"[MeSH Terms] OR "urothelial carcinoma"[tiab] OR "bladder cancer"[tiab]
  OR "carcinoma, renal cell"[MeSH Terms] OR "renal cell carcinoma"[tiab]
  OR "urogenital neoplasms"[MeSH Terms] OR "genitourinary cancer"[tiab])
AND
("treatment selection"[tiab] OR "predictive biomarker"[tiab] OR "resistance monitoring"[tiab]
  OR "treatment response"[tiab] OR "neoplasm, residual"[MeSH Terms] OR "minimal residual disease"[tiab])
Filters: 2020/01/01–2026/04/30; English
```

2. Embase (Ovid syntax)

```
1. 'circulating tumor DNA'/exp OR 'circulating tumor DNA':ti,ab OR 'ctDNA':ti,ab
   OR 'liquid biopsy':ti,ab OR 'cell free DNA':ti,ab
2. 'prostate cancer'/exp OR 'prostate cancer':ti,ab
   OR 'bladder cancer'/exp OR 'urothelial carcinoma':ti,ab OR 'bladder cancer':ti,ab
   OR 'kidney cancer'/exp OR 'renal cell carcinoma':ti,ab
   OR 'urogenital tumor'/exp OR 'genitourinary cancer':ti,ab
3. 'treatment selection':ti,ab OR 'predictive biomarker':ti,ab OR 'resistance monitoring':ti,ab
   OR 'treatment response':ti,ab OR 'minimal residual disease'/exp OR 'minimal residual disease':ti,ab
4. 1 AND 2 AND 3
5. limit 4 to (english language AND yr="2020-2026")
```

3. Cochrane Central Register of Controlled Trials (CENTRAL)

```
#1  MeSH descriptor: [Circulating Tumor DNA] explode all trees
#2  ("circulating tumor DNA" OR "ctDNA" OR "liquid biopsy" OR "cell-free DNA"):ti,ab,kw
#3  #1 OR #2
#4  MeSH descriptor: [Prostatic Neoplasms] OR [Urinary Bladder Neoplasms]
```

OR [Carcinoma, Renal Cell] OR [Urogenital Neoplasms] explode all trees
#5 ("prostate cancer" OR "urothelial carcinoma" OR "bladder cancer"
OR "renal cell carcinoma" OR "genitourinary cancer"):ti,ab,kw
#6 #4 OR #5
#7 ("treatment selection" OR "predictive biomarker" OR "resistance monitoring"
OR "treatment response" OR "minimal residual disease"):ti,ab,kw
#8 #3 AND #6 AND #7
Publication Year from 2020 to 2026, in Trials

4. Web of Science (Core Collection)

TS=("circulating tumor DNA" OR "ctDNA" OR "liquid biopsy" OR "cell-free DNA")
AND
TS=("prostate cancer" OR "urothelial carcinoma" OR "bladder cancer"
OR "renal cell carcinoma" OR "genitourinary cancer")
AND
TS=("treatment selection" OR "predictive biomarker" OR "resistance monitoring"
OR "treatment response" OR "minimal residual disease")
Refined by: Publication Years 2020-2026; Language: English
